# Investigation of an airport-associated cluster of falciparum malaria in Frankfurt, Germany, 2022

**DOI:** 10.2807/1560-7917.ES.2024.29.5.2300298

**Published:** 2024-02-01

**Authors:** Johanna Kessel, Anna Rosanas-Urgell, Tobias Dingwerth, Udo Goetsch, Jonas Haller, Ralph Huits, Johanna H Kattenberg, Anna Meinecke, Pieter Monsieurs, Michael Sroka, Torsten Witte, Timo Wolf

**Affiliations:** 1 Goethe University, University Hospital Frankfurt, Department of Infectious Diseases, Frankfurt, Germany; 2 Unit of Malariology, Department of Biomedical Sciences, Institute of Tropical Medicine, Antwerp, Belgium; 3 Municipal Health Protection Authority, Frankfurt, Germany; 4 Hannover Medical School, Department of Rheumatology and Immunology, Hannover, Germany; 5 Goethe University, Department of Integrative parasitology and animal physiology, Frankfurt, Germany; 6 Fraport AG, Medical Services, Frankfurt, Germany; 7 Medical Center Frankfurt, Medical Services & Health Management Lufthansa Group, Frankfurt, Germany; 8 Department of Infectious Tropical Diseases and Microbiology, IRCCS Sacro Cuore Don Calabria Hospital, Negrar di Valpolicella, Verona, Italy

**Keywords:** airport, malaria, molecular analysis, imported infectious diseases, emerging infectious diseases

## Abstract

Airport malaria is uncommon but increasing in Europe and often difficult to diagnose. We describe the clinical, epidemiological and environmental investigations of a cluster of airport malaria cases and measures taken in response. Three Frankfurt International Airport employees without travel histories to malaria-endemic areas were diagnosed with *Plasmodium falciparum* malaria in Germany in 2022. Two cases were diagnosed within 1 week, and the third one after 10 weeks. Two cases had severe disease, all three recovered fully. The cases worked in separate areas and no specific location for the transmissions could be identified. No additional cases were detected among airport employees. In June and July, direct flights from Equatorial Guinea, Nigeria and Angola and one parcel originating in Ghana arrived at Frankfurt airport. No vector-competent mosquitoes could be trapped to identify the source of the outbreak. Whole genome sequencing of *P. falciparum* genomes showed a high genetic relatedness between samples of the three cases and suggested the geographical origin closest to Ghana. A diagnosis of airport malaria should prompt appropriate and comprehensive outbreak investigations to identify the source and to prevent severe forms of falciparum malaria.

**Key public health message**

**What did you want to address in this study?**

Malaria is a serious disease in humans, caused by a parasite and transmitted by mosquitoes. In Europe, most malaria cases are related to travel to countries where the disease is common. In 2022, three persons working at Frankfurt Airport developed malaria. We describe the outbreak investigation and the measures taken in response to the cluster of cases.

**What have we learnt from this study?**

A diagnosis of airport malaria should prompt appropriate and comprehensive outbreak investigations possibly using molecular methods. In this cluster, two of three patients had severe disease. One case could only be diagnosed very late.

**What are the implications of your findings for public health?**

Health professionals should be aware of the possibility of airport malaria. Diagnostic procedures for patients with fever working at or residing near airports should be adapted in non-endemic countries in specific periods, e.g. May to October in Central Europe. Results of investigations can inform prophylactic measures.

## Background

Malaria is a parasitic disease caused by *Plasmodium* species and affects an estimated 247 million patients in 84 countries worldwide [1]. Since 1974, Europe has been considered malaria-free, and in 1964 the World Health Organization (WHO) certified Germany free of indigenous malaria [2]. Today, the overwhelming majority (99.8%) of the 8,000–9,000 annual cases in Europe are travel-associated and the yearly incidence rate is 1.2 cases per 100,000 inhabitants [3]. In Germany, where malaria is a notifiable disease, 999 cases were reported in 2019. In Europe, the notified cases decreased significantly during the coronavirus disease 2019 (COVID-19) pandemic (366 notified cases in 2020) [3]. After the travel restrictions were lifted, an increase of severe malaria cases was reported [4], the reason of which remains unclear to date.

Autochthonous malaria transmission can occur when the parasite and a competent vector population meet. This was demonstrated in 2011 in Greece in a *P. vivax* outbreak linked to seasonal workers from malaria-endemic countries [5,6]. Non-imported malaria in non-endemic areas can occur congenitally, via blood transfusions or organ transplants and at airports [7]. Potential modes of transmission are either by endemic mosquitoes of *Anopheles* species with ingested gametocytes from carriers or imported *Plasmodium*-infected mosquitoes (Odyssean malaria) [8].

Tracing the origins of airport malaria can be challenging if there is either no direct airport connection [9] or it is the only risk factor for transmission [10]. Recent cases caused by imported *Anopheles* species in the vicinity of airports did not lead to further transmission [11,12]. However, airport malaria has increased in Europe [13]. Epidemiological modelling indicates that in the decades to come, a spread of vector-competent species northwards and a possible re-emergence of autochthonous malaria in southern Europe is likely due to climate change [14,15].

As malaria is mainly a tropical disease, without relevant travel histories, the diagnosis can be missed. To prevent adverse or fatal outcomes, a rapid diagnosis is essential [11].

## Outbreak detection

On 12 and 13 July 2022, within 24 hours, two employees at Frankfurt Airport without a history of travel to malaria-endemic areas were diagnosed with *P. falciparum*. A third airport employee was diagnosed on 14 September, 70 days after onset of fever. The outbreak of airport malaria was reported to the Frankfurt Health Authority on 13 July. An investigation of the events, including a search for possible further cases, was initiated together with the airport operator, the biggest airline and the medical services.

Here we describe the cluster of airport-associated malaria as including molecular, epidemiological and environmental investigations were performed to identify a single or multiple sources for the infections and to understand the transmission at the airport.

## Methods

### Case investigations

The three cases who tested positive for malaria, were interviewed by infectious disease and public health specialists on the clinical symptoms, onset of illness, the job tasks at the airport, e.g. transport of luggage, boarding of airplanes, cargo halls, working hours (day/night shift) and recalled insect bites. Diagnostics such as peripheral blood counts, thin blood smears as well as quantitative buffy coat (QBC) fluorescent microscopy and clinical chemistry tests were performed as part of the clinical routine. Severe malaria was diagnosed according to the German guidelines [16]. Clinical data were extracted from files.

### Epidemiological investigations

Employees at Frankfurt Airport were informed via a globally accessible intranet service, the app Beekeeper (https://www.beekeeper.io/) and by phone about the occurrence of airport malaria in Frankfurt. They were asked to contact healthcare services in case of fever > 38°C and other symptoms, such as headache, muscle pain or diarrhoea. Employees in the cargo area who were on sick leave and co-workers of the first two patients were personally contacted.

Surveillance data from the federal state of Hesse, where Frankfurt is situated, and from all bordering districts to Hesse that were residential areas of airport employees were searched for malaria cases with no travel history by direct enquiry of the Frankfurt municipal health office to the Robert Koch Institute (RKI), the national public health institute. Schedules of direct flights between malaria-endemic areas and Frankfurt between 15 and 30 June were screened, based on a 21-day incubation period of malaria. In addition, cargo deliveries with origin in Ghana, were checked.

### Entomological investigations

An entomological investigation in and around the main airport cargo hall and the work areas of the forklift driver was carried out. The surrounding area with a perimeter of 100 m was inspected for stagnant water and potential breeding sites, based on the presumption that a potential vector could have been introduced by cargo, similarly to earlier cases of airport malaria in Frankfurt [12]. Mosquito traps (BG-Sentinel Biogents, Regensburg, Germany) baited with BG Lure and connected with CO_2_ gas cylinders were set out. Four traps were placed on 15 July 2022 and inspected daily for 2 weeks.

### Molecular investigations of *Plasmodium falciparum*

At Frankfurt University Hospital, a ca 5 mL sample of venous blood in EDTA was obtained before initiation of antimalarial treatment and sent to Unit of Malariology of the Institute of Tropical Medicine, Antwerp, Belgium for analysis. At the Unit of Malariology, DNA was extracted with QIAmp DNA mini blood kit (Qiagen, Venlo, the Netherlands) following the manufacturer’s instructions. Extracted DNA was eluted in 200 µL elution buffer. A selective whole genome amplification was applied to 30 µL of the extracted DNA as previously described [17] and the resulting DNA was sent to GenomeScan (Leiden, the Netherlands) for sequencing.

We generated 2 × 150 base pairs (bp) paired sequencing reads (PE) on the NovaSeq platform (Illumina, San Diego, the United States). The sequencing data are available at the National Center for Biotechnology Information (NCBI) Short Read archive (https://www.ncbi.nlm.nih.gov/sra/docs/) under accession number PRJNA955667. The *P. falciparum* Community Project (Pf6) whole genome sequencing (WGS) was used in the analysis [18], (https://www.malariagen.net/). The outbreak genomes were aligned to the reference sequence and variants were called in a similar pipeline as the Pf6 WGS data as previously described [11]. All genetic variants used in the previous study were combined with data from our outbreak investigation using the merge command of the BCFtools (binary variant call format tools) and biallelic single nucleotide polymorphism (SNP) variants in the core genome region only were filtered [19].

The geographical origin of the *P. falciparum* isolates was explored using a principal component analysis (PCA) as implemented in the scikit-allele python module and using a discriminant analysis of principal components (DAPC) from the adegenet package in R [20,21], following methods previously described [11]. The PCA was applied to two datasets: (i) *Plasmodium* samples from all countries which passed quality control and (ii) isolates from West and Central Africa. Then Discriminant Analysis of Principal Components (DAPC) was used to calculate the discriminant components. Based on the DAPC results, posterior probabilities were calculated, which gives the probability for each country whether either of the samples has a similar genetic profile as the isolates from the countries in the reference dataset.

The relatedness between our isolates and samples nearest in the DAPC plot and posterior prediction (Ghana, Guinea, Mali, Nigeria and Benin) was subjected to identity-by-descent (IBD) analysis, which indicates common ancestry between strains, using the R package isoRelate [22]. Pairwise relatedness mapping was done with the genomes, which uses a first order continuous time hidden Markov model allowing for multiple infections. Pairwise comparisons between isolates from Germany and countries in the identified cluster were conducted, and the proportion of the genome sharing IBD calculated. Known drug and diagnostic resistance markers were identified through bioinformatic analysis pipelines previously set up [23].

## Results

### Case characteristics

#### Case 1

A bus driver in their 50ies at Frankfurt airport developed fever, diarrhoea and a dry cough on 5 July 2022 and went to hospital on 7 July. Comorbidities were hypertension, coronary heart disease, obesity (Body Mass Index: 37; normal range: 18.5-24.9), steatosis hepatis and heterozygous sickle cell anaemia. Case 1 had not travelled to malaria-endemic areas, not noticed any insect bites and not received blood transfusions in the past. Seven days after starting empirical antimicrobial therapy, malaria parasites were detected in thin blood smear.

Case 1 was transferred to Frankfurt University Hospital, where *P. falciparum* was confirmed at a parasite density of 0.15%. (Table). Case 1 did not fulfil the criteria for complicated malaria and was treated with a combination of artemether and lumefantrine (80/480 mg) orally (p.o.) at 0, 8, 24, 36, 48 and 60 hours and became afebrile after 36 hours and QBC fluorescent microscopy was negative at 48 hours. Three days later, fever reoccurred, and recrudescence was found in a thin blood smear after which Case 1 was treated with a combination of atovaquone and proguanil (1,000/400 mg p.o. once a day for 3 days). All further controls remained negative. Case 1 made a full recovery.

**Table:** Characteristics and laboratory findings in a cluster of malaria cases at Frankfurt airport, Germany, July–September 2022 (n = 3)

Characteristics	Case 1	Case 2	Case 3	Reference range
Age group (years)	50 to 59	30 to 39	40 to 49	Not applicable
Occupation	Bus driver	Forklift driver	Security worker	
Time from symptom onset to diagnosis (days)	7	7	70	
Altered consciousness	No	**Yes**	**Yes**	
Respiratory insufficiency^a^	Yes (oxygen supplement 1L/min)	Yes (high flow therapy, FiO2 40%, 25L/min)	No	
Hypotension (RRsys < 90 mmHg or MAP < 65 mmHg)	No	No	No	
Signs of bleeding	No	No	No	
Oliguria (< 400 mL/24 h)	No	No	No	
Baseline parasitaemia (%)	0.15	**7**	**15**	0
Haemoglobin (g/dL)	11.5	12.9	**6.8**	13.5 to 17.5
Leucocytes (/nL)	7.18	17.68	2.4	3.92 to 9.81
Platelets (/nL)	38	24	26	146 to 328
CRP (mg/dL)	12.67	16.76	4.74	< 0.50
Sodium (mmol/L)	138	125	134	135 to 145
Potassium (mmol/L)	3.5	4.6	3.6	3.6 to 4.8
Bicarbonate (mmol/L)	NA	20.8	23	21 to 26
pH	NA	7.46	7.38	7.35 to 7.45
Lactate (mg/dL)	NA	34	35.13	4.5 to 20.0
Base excess	NA	-5.5	-2.0	-2 to 3
Blood glucose (mg/dL)	160	94	153	74 to 106
Bilirubin (mg/dL)	0.9	2.1	2.98	< 1.4
ALAT (U/L)	94	294	49	< 50
ASAT (U/L)	73	658	60	< 40
GGT (U/L)	NA	84	123	< 60
LDH (U/L)	449	1,485	769	< 248
Creatinine (mg/dL)	1.29	1.58	0.56	0.70 to 1.20
Urea (mg/dL)	NA	94	49	18 to 55

ALAT: alanine transaminase; ASAT: aspartate aminotransferase; CRP: C-reactive protein; FiO2: fraction of inspired oxygen; GGT: gamma-glutamyltransferase; LDH: lactate dehydrogenase; MAP: mean arterial pressure; mmHg: millimetre of mercury; NA: not analysed; RRsys: systolic blood pressure; U: enzyme unit.

^a^ O_2_-saturation < 90% at room air or O_2_ suppletion to maintain saturation > 95% or endotracheal intubation and mechanical ventilation.

Values representing a criterion for severe malaria are shown in bold.

#### Case 2

A forklift driver in their 30ies at Frankfurt Airport developed fever on 6 July. On 13 July, Case 2 was referred to Frankfurt University Hospital by the intensive care unit of another hospital after presenting with fever, respiratory insufficiency, altered consciousness, prostration, metabolic acidosis with hyperlactataemia and acute kidney injury (Table). Case 2 had not travelled to malaria-endemic areas and did not recall any insect bites.

The transferring hospital reported a positive malaria rapid antigen detection test, *P. falciparum* was confirmed in a thin blood smear with a parasitaemia of 7% and complicated malaria was diagnosed (Table). A computer tomography (CT) scan and magnetic resonance imaging (MRI) of the brain did not show pathologies. A chest radiograph showed prominent pulmonary vessels, pleural effusions and consolidations, not convincingly corresponding to pulmonary oedema.

Case 2 received treatment with intravenous artesunate (4 × 2.4 mg/kg at 0, 12, 24 and 48 h until parasite clearance), followed by atovaquone-proguanil (1,000 mg/400 mg p.o. once a day for 3 days) and was discharged on 21 July. A self-limiting haemolysis was noted 9–16 days after the initiation of artesunate. Case 2 made a full recovery.

#### Case 3

A security service worker in their 40ies at Frankfurt Airport developed fever and malaise on 6 July. Case 3 was diagnosed with a severe *P. falciparum* infection on 14 September. Case 3 had no travel history to malaria-endemic areas or blood transfusion in the past.

Initially, they presented to hospital on 10 July 2022 but remained without a diagnosis and had nine pairs of negative blood cultures. A chest radiography and an ultrasound of the abdomen showed hepatosplenomegaly. Case 3 received empirical antimicrobial treatment starting on 12 July with amoxicillin-clavulanate (875/125 mg twice a day p.o. for 7 days), piperacillin-tazobactam (4.5 g three times a day intravenously (i.v.) for 7 days) and ciprofloxacin (500 mg twice a day p.o. for 7 days), meropenem (1 g three times a day i.v. for 6 days) and vancomycin (1 g twice a day i.v. for 6 days). Case 3 developed mild apraxia and sensory ataxia but an MRI of the brain showed no abnormalities. White cell pleocytosis (18/µL) was observed. In August, an autoimmune disorder with a secondary hemophagocytic lymphohistiocytosis (HLH, a rare systemic inflammatory syndrome) was suspected as six of the eight diagnostic criteria were present [24] and Case 3 was treated with 500 mg prednisolone, followed by 10 mg/kg dexamethasone. Subsequently, C-reactive protein (CRP) decreased from 77.9 mg/L to 9.2 mg/L while haemoglobin and platelets improved (from 7.1 g/dL to 8.8 g/dL and from 143 k/µL to 230 k/µL, respectively). After steroid dose reduction, Case 3 relapsed and needed intensive care treatment due to hypotension and confusion. Triggered by haemolytic anaemia, a thin blood smear was performed on 14 September and *P. falciparum* was detected at a parasitaemia of 15% (Table). Case 3 received artesunate (2.4 mg/kg i.v., five doses until parasite clearance at 0 h, 12 h, 24 h, 48 h and 72 h), followed by atovaquone-proguanil (1,000/400 mg p.o. once a day for 3 days). Case 3 became afebrile, was discharged 6 days after the diagnosis and made a full recovery.

### Epidemiological findings

Case 1 had been on night duty 1–8 days before symptom onset and Case 2 9–14 days before. Based on a minimum incubation period of 7 days and by combining the dates for onset of symptoms with the work schedules, we suspect that the transmission from the mosquito occurred 27–28 June. All three cases were working early shifts starting at 5 AM 27–30 June. Cases 1 and 2 worked in the vicinity of arriving aircrafts and Case 3 worked as a security agent at access gates. All three worked in different areas and did not know each other. They did not share workspaces, canteens or changing rooms. Cases 1 and 2 worked in areas that were 2.9 km apart. The bus driver (Case 1) circulated the airport, passed by the security area and the cargo hall and stopped 600 m away from the cargo hall, opening the doors briefly at both stops.

**Figure 1:**
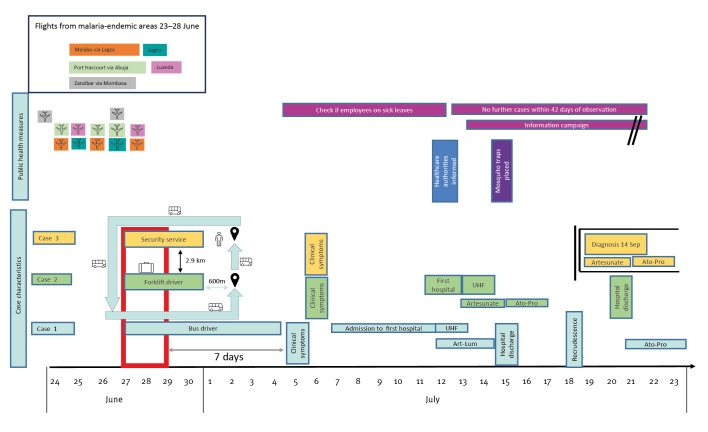
Case characteristics, investigations and response measures taken in a cluster of malaria cases at Frankfurt airport, Germany, June–September 2022 (n = 3)

Duty rosters of the three cases showed no assignment to arriving aircrafts or handling of cargo from malaria-endemic areas. At Frankfurt Airport, flights arrive from only a few destinations of malaria-endemic areas. Between 23 and 28 June, there were direct flights from Nairobi (Kenya), Mombasa (Kenya), Malabo (Equatorial Guinea) with a stop-over in Lagos (Nigeria), Port Harcourt via Abuja (Nigeria) and non-stop flights to Luanda (Angola) (Figure 1). Checking cargo waybills of Lufthansa Cargo showed two parcels originating in malaria-endemic areas: one from Accra (Ghana) transported by road from Brussels to Frankfurt and arriving there on 29 June and another from Abidjan (Côte d’Ivoire).

Art-Lum: artemether-lumefantrine; Ato-Pro: atovaquone- proguanil; UHF: Frankfurt University Hospital.

Case 1 is indicated in pale blue, Case 2 in green and Case 3 in yellow.

The red box marks the likely period of transmission based on the work rosters of the cases.

### Molecular findings

**Figure 2:**
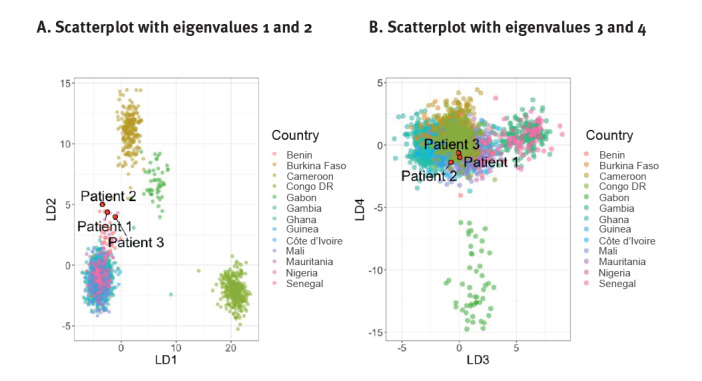
Scatter plot of discriminant analysis of principal components of *Plasmodium falciparum* genomes from West and Central Africa and cases of airport-associated malaria in Frankfurt, Germany (n = 2,625)

We obtained *P. falciparum* whole genome sequences from samples of the three cases (274,818–12,151,560 PE reads resulting in 36–159X coverage). When comparing these genomes with a database of *P. falciparum* genome sequences from across the world, the genomes of the three isolates cluster with isolates from West and Central Africa in the PCA, based on the genetic variation observed in those genomes. Supplementary Figure 1 shows the results of a PCA analysis of the first four principal components around the world and in Africa. The outbreak isolates were placed in the large cluster with isolates from West and Central Africa. Using DAPC, the between-country variation was analysed, and our isolates were positioned closest to Benin, Nigeria and Ghana (Figure 2) and were predicted with the highest posterior probability (0.95 < p < 0.99) to belong to a cluster with sequences from Ghana, followed by Mali (0.0056 < p < 0.048).

Panel A. Scatter plot of discriminant analysis (LD) eigenvalues 1 and 2 shows clustering of the cases near a cluster of genomes from Western Africa, closest to an outgroup of samples from Nigeria and Senegal.

Panel B. Scatter plot of LD eigenvalues 3 and 4 groups the samples at the centre of a larger cluster of western African isolates. DAPC plotted in R.

Genomic analysis of the *P. falciparum* isolates from the three cases showed that the parasites were highly related (IBD > 95%). The first and the third sample showed 99% IBD, while the second had 96% IBD compared with the other two, indicating that the three cases were infected with the same parasite strain. Network analysis of IBD adds evidence to the prediction of origin in Ghana (Figure 2).

We investigated variants in genes associated with drug resistance, e.g. *kelch 13, crt, mdr1*, in the three parasite genomes and found no validated markers of resistance.

### Outbreak response

In response to the outbreak, an active case finding was initiated. Employees at Frankfurt airport received information and education by the airport operator and one of the major airlines within the following 42 days after the first case was detected. This long follow-up period was chosen prospectively as it was unclear for how long transmission could be expected to have occurred. Employees with symptoms compatible with malaria were prompted to contact healthcare services. No additional malaria cases were identified among the employees or individuals living in the vicinity of the airport during the six weeks after notification of the first cases.

The cargo hall was inspected for potential breeding sites. No stagnant water as a potential breeding site for mosquitoes could be found. Traps were placed on 15 July 2022, 3 days after the first patient was diagnosed (Figure 1). No *Anopheles* mosquitoes could be trapped. One mosquito, visually identified as *Culex*, was trapped but escaped. Thus, these results did not allow to identify the specific location of malaria transmission.

## Discussion

We detected an outbreak of malaria among three employees at Frankfurt Airport, which was the second cluster of airport malaria in Frankfurt within 5 years [12] and represents an unusual event. Two of the three cases had severe malaria. In contrast to the earlier report of airport malaria, the affected employees worked at different locations of the airport, the only connection was through Case 1, who drove a bus between the workplaces of Case 2 and 3, but we could not demonstrate that mosquitoes were translocated by that bus. *Anopheles* may have a flight range of up to 7 km under favourable weather conditions [25], so a single mosquito could have covered the distance. Furthermore, no possible breeding sites were identified. Altogether, no location for a common transmission event could be identified, but molecular analysis showed a likely transmission by a single mosquito despite the different work locations. No further suspected or confirmed cases were notified.

The third case was diagnosed 70 days after symptom onset, which reflects the difficulty of making this diagnosis in a non-endemic country without knowing the epidemiological link. This led to important delays in adequate diagnosis and treatment. If the healthcare professionals had asked about the workplace or considered malaria as a possibility in an airport employee, an earlier diagnosis could have been probable. Prompt recognition and adequate management of *P. falciparum* infections can avert severe malaria. Blood smears and specific testing, such as rapid antigen testing, should be used in febrile patients, living or working in the vicinity of an airport. Communication to increase awareness of the increasing incidence of vector-borne diseases for the general public, but especially for health professionals, is necessary.

One case experienced recrudescence with no resistance mutations detected. The high body weight of the case may have weakened the effect of the treatment and artemisin-based medications may have to be given for a longer time to be fully effective.

A likely period for transmission could have been 7 days before the onset of symptoms during the common work schedule of the patients. This may mean that the transmission occurred 27–28 June. The molecular analysis predicted Ghana as the country of origin, but there were no direct flights from Ghana during the period of vector importation. A parcel from Ghana was handled in the cargo hall, however, but it arrived on 29 June, which would mean that if this was the source, the incubation period would have been extremely short with 6 days for one case and 7 days for the other two.

We could not catch any *Anopheles* species from the airport. Only one mosquito was trapped, identified as *Culex* species. However, the traps were placed 18 days after the suspected transmission. In the future, a regular use of mosquito traps during the summer period should be considered. The high genetic relatedness of *P. falciparum* isolates from the three cases indicates that they were infected with a single parasite strain, i.e. one mosquito could have infected all three persons. This is strongly supported considering the high level of transmission and genetic diversity of parasites in West Africa, an IBD > 95%, which is nearly clonal, and a symptom onset within a time window of less than 48 hours. Despite the fact that the origin was predicted to be in Ghana, we could not determine a direct flight or cargo material coming from this country at a convincingly relevant time. This could be explained by a high level of genetic flow in West African countries and that not all West African sequences are represented in the public databases.

Summer 2022 was one of the warmest in Germany since the meteorological measurements began [26]. In the suspected transmission period of 27–28 July, the recorded temperatures peaked at 25°C and 27°C, respectively (minimum night temperature was 13.6°C on 27 July and 11.6°C on 28 July, average temperatures 20°C and 20.5°C). An increasing frequency of unusually hot summers may enhance the survival of imported, exotic *Anopheles*, which may then be active enough to ingest a blood meal and pass on *Plasmodia*. Vector-competent *Anopheles* are present in many European countries [27]. International traffic may also promote the spread of vector-borne diseases [28,29].

This report has limitations and there were challenges in the response. The investigations could only be started late based on the discovery of the index cases. Furthermore, clinical data were collected retrospectively and not all information was available. There were gaps in the early medical history of Case 3 in particular as many caregivers were involved. No *Anopheles* species could be trapped, even though the trapping system was validated for the species [30] and drainage holes at relevant sites could not be controlled. Predicting the country of origin from genomic data depends on the completeness of the databases including geographical representation and time difference between collection of reference datasets and isolates. The parasite population may change over time by frequent recombination and high levels of admixture. Establishing networks among research institutes and reference laboratories in endemic and non-endemic regions, in addition to data-sharing platforms, would expand existing datasets and thereby improve the accuracy of tracing.

## Conclusions

Over the past few years, Europe has experienced a rise in imported cases of malaria, a development which could be accelerated by climate change. Our results underscore the utility of genomic surveillance for unravelling transmission chains and predicting importation routes. Airport staff and medical personnel should receive more training on of the possibility of non-endemic diseases by specialists in infectious diseases and in public health, to improve awareness. The use of malaria rapid tests or other established diagnostic methods for international airport workers displaying malaria-like symptoms should be integrated into the diagnostic work-up during relevant periods like May to October. Mosquito traps, regularly set at different locations of airports during summer, could help monitor imported mosquitos.

## Supplementary Data

538000 Supplementary Material
