# Supplementary material for: Investigation of an airport-associated cluster of falciparum malaria in Frankfurt, Germany, 2022
Source: Euro Surveill. 2024 Feb 1;29(5):2300298. doi: 10.2807/1560-7917.ES.2024.29.5.2300298 (PMC10835754; doi:10.2807/1560-7917.ES.2024.29.5.2300298)
Supplement: Supplementary Material [file 23-00298_WOLF_Supplement.pdf]

This supplementary material is hosted by Eurosurveillance as supporting information alongside the article *Investigation of an airport-associated cluster of falciparum malaria in Frankfurt, Germany, 2022*, on behalf of the authors, who remain responsible for the accuracy and appropriateness of the content. The same standards for ethics, copyright, attributions and permissions as for the article apply. Supplements are not edited by Eurosurveillance and the journal is not responsible for the maintenance of any links or email addresses provided therein.

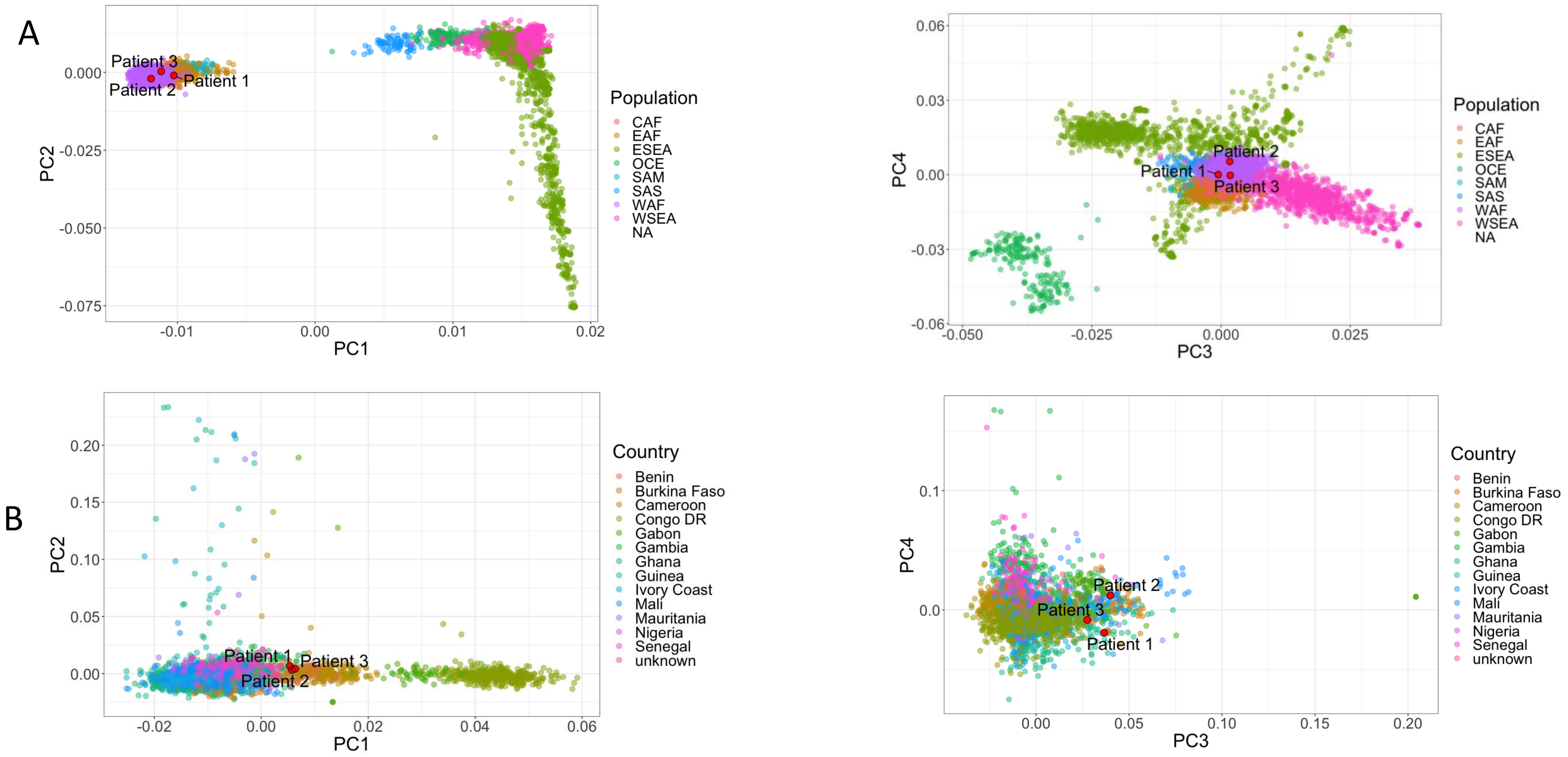

Supplementary Figure 1.Principle component analysis (PCA) of biallelic variants in the core genome region of isolates from A) across the whole world, and B) African countries. The first two components are depicted on the left, components 3 and 4 on the right of the figure. Isolates collected in Germany are indicated as red dots, and cluster with isolates from Western Africa. WAF = West Africa, EAF = East Africa, WSEA = West South-East Asia, ESEA = East South-East Asia, OCE = Oceania, SAM = South America, SAS = South Asia, CAF = Central Africa.
